# Staples Versus Sutures for Skin Closure in Standard Four Port Laparoscopic Cholecystectomy: A Prospective Cohort Study

**DOI:** 10.7759/cureus.13725

**Published:** 2021-03-06

**Authors:** Farhanul Huda, Bhargav Gajula, Sudhir Singh, Shashank Kumar, Manoj Joshua Lokavarapu, Durga Sowmya

**Affiliations:** 1 General Surgery, All India Institute of Medical Sciences, Rishikesh, IND

**Keywords:** laparoscopy, cholecystectomy, skin suture, staples, wound infection

## Abstract

Introduction

Many studies have been done comparing sutures versus skin staples in various wounds. To the author's knowledge, there is no study comparing these two in an laparoscopic cholecystectomy (LC) wound. Our study aims at comparing the clinical outcome of skin closure by monofilament nylon suture and stainless-steel skin stapler in standard four-port LC. The results of this study can help in developing guidelines for skin closure in LC.

Objective

To compare the clinical outcome of skin closure by monofilament nylon suture and stainless-steel skin stapler in standard four-port LC.

Methods

The study was conducted as a time-bound prospective cohort study on diagnosed patients of cholelithiasis admitted in a single unit of the Department of Surgery at All India Institute of Medical Sciences, Rishikesh, India from February 2018 to February 2019. The standard four-port LC was done by the same surgeon. After the completion of the surgery, port closure was done using absorbable sutures, and skin was closed by either 2.0 monofilament nylon suture (Ethilon, Ethicon, Scotland) or stainless-steel staples (Proximate plus MD, 35W, Ethicon, Scotland). The time taken for skin closure in both the groups was noted using a stopwatch. Each wound was assessed on the post of day (POD) 1 during discharge, on POD 10 during suture/stapler removal, and POD 30 by the operating surgeon for pain, wound infection, scar status using validated scales.

Statistical analysis used

The outcome measures were calculated as mean and standard deviation. Continuous variables were analyzed using a two-tailed student t-test.

Results

Out of 48 suture vs 45 stapler cases the average time for closure is 277.14 seconds in suture vs 77.2 seconds (p = 0.0001) in the stapler group. All other parameters studied were not statistically significant among the two cohorts.

Conclusion

We conclude that stapler requires minimum time for closure with no statistically significant difference in wound infection, post-op pain, pain during removal, and scar results are the same in both the groups.

## Introduction

Laparoscopic cholecystectomy (LC) is the gold standard treatment for gallstone disease and is the most common laparoscopic surgery performed worldwide [1]. Surgical site wound closure plays a very important role in the postoperative success of any surgery. This importance is magnified with regards to LC in obtaining cosmesis. Wound complications are one of the most common causes of morbidity and mortality in laparoscopic procedures. Surgical wound closure aims to achieve rapid wound healing and a satisfactory cosmetic result, and also to reduce the risks of complications. Despite the large number of LC performed there is no agreed standard on the skin closure. Different methods and materials are used for wound closure. Skin closure of laparoscopic port entry wounds in LC is usually done with sutures. Staples are an alternative option to sutures. The potential advantage of staples is their low level of tissue reactivity thus reducing the local inflammatory response. It also reduces the width of the wound, time taken for wound closure, and the residual cross marks [2].

Many studies have been done comparing sutures versus skin staples in various wounds. To the author's knowledge, there is no study comparing these two in an LC wound. Our study aims at comparing the clinical outcome of skin closure by monofilament nylon suture and stainless steel skin stapler in standard four-port LC. The results of this study can help in developing guidelines for skin closure in LC.

## Materials and methods

The study was conducted as a time-bound prospective cohort study on diagnosed patients of cholelithiasis admitted in a single unit of the Department of Surgery at All India Institute of Medical Sciences, Rishikesh from February 2018 to February 2019. Informed consent was taken from each patient and the patients were randomized into the suture and the stapler group by computer randomization method. Each patient was screened as per the inclusion and exclusion criteria.

Patients with age >18 years and <80 years without comorbidities undergoing elective LC are included in the study. Patients with acute calculus cholecystitis, empyema gall bladder, immunocompromised patients, and LCs converted to open procedures were excluded from the study.

Standard preoperative preparation was followed in both the study groups. The LC was completed by the standard four-port technique by the same surgeon. After the completion of the surgery, the port closure was done in layers by absorbable sutures till the subcutaneous layer, and the skin was closed by either 2.0 monofilament nylon sutures (Ethilon, Ethicon, Scotland) by simple interrupted fashion, two stitches for 10 mm port and one stitch for 5 mm port or stainless-steel staples (Proximate plus MD, 35W, Ethicon, Scotland), three staples for 10 mm port and one staple for 5 mm port as per randomization. Each patient was given an I/V antibiotic according to the SAGES guidelines (single preoperative dose given within one hour of skin incision, and re-dosed if the procedure is more than 4 hours long). The time taken for skin closure in both groups was noted using a stopwatch. Each wound was assessed on the post of day (POD) 1 during discharge, on POD 10 during suture/stapler removal, and on POD 30 by the operating surgeon for pain, wound infection, and scar status using validated scales.

Outcomemeasures

Time taken for skin closure, rate of wound infection assessed by Southampton Grade [3], pain during suture\staple removal on POD1, 10, and 30 were evaluated by a numeric rating scale [4], the post-op scar was evaluated using the Stony Brook scar evaluation scale (SBSES) [5], rate of readmission, the requirement of secondary procedures like wound debridement, wound wash and resuturing were assessed for outcome measures.

Statistical analysis

Data was collected by a trained surgery resident and entered into an electronic database (MS Excel, Microsoft, Redmond, WA) and analyzed using statistical software SPSS (IBM, Armonk, NY, USA). The outcome measures were calculated as mean and standard deviation. Continuous variables were analyzed using a two-tailed student t-test. The results were reported as per the guidelines of the STROBE (Strengthening the Reporting of Observational Studies in Epidemiology) group [6].

## Results

Age

The mean age of the suture group was 39.75 and that of the stapler was 38.4. The difference was not statistically significant with a p-value of 0.613. Hence the study group was comparable in terms of age.

Gender

The majority of the study population were females comprising 79.57% and the rest 20.43% were males.

Closure technique

A total of 48 patients underwent skin suturing technique whereas 45 underwent stapler closure.

The technique of wound closure and meantime of closure

The mean time of closure in the suture group was 277.14 seconds and in the stapler group was 77.2 seconds. The difference was found to be statistically significant with P < 0.001; (95 CI - 166.5 to 233.3) (Table 1).

**Table 1:** Results of mean time for wound closure among suture and stapler group.

Group	N	Mean Time (seconds)	Standard Deviation (SD)	T-value	P-value
Suture	48	277.1	96.9	1.618	0.0001
Stapler	45	77.2	59.67		

The technique of wound closure and surgical site infection

Among the suture group, 83.3% and 95.8% had Grade 0 on POD1 and POD 30, respectively. Among the stapler group, 93.7% and 97.7% had Grade 0 on POD1 and POD30, respectively. None of the patients developed Grade 2 surgical site infection (SSI) in the stapler group and 8.3% of patients developed Grade 2 SSI in the suture group. However, these results were not statistically significant (Table 2).

**Table 2:** Results of surgical site infection among suture and stapler group. *n = 44; four patients were lost to follow-up on POD 10.

Group	Southampton Grade on Post Operative Day (POD)	Normal Healing (Grade 0), n (%)	Normal Healing with Mild Erythema (Grade 1), n (%)	Erythema+ Other Signs of Inflammation (Grade 2), n (%)	Serous Discharge or Pus (Grade 3 and 4), n (%)
Suture (n = 48)	POD1	40 (83.3)	8 (16.6)	0 (0)	0 (0)
	POD10*	31 (64.5)	9 (18.7)	4 (8.3)	0 (0)
	POD30	46 (95.8)	2 (4.1)	0 (0)	0 (0)
Stapler (n = 45)	POD1	42 (93.7)	3 (6.6)	0 (0)	0 (0)
	POD10	40 (88.8)	5 (11.1)	0 (0)	0 (0)
	POD30	44 (97.7)	1 (2.2)	0 (0)	0 (0)

Type of closure technique and numeric rating scale

The mean numeric rating scale of the suture group was 1.64 and 1.10, for the stapler group it was 1.10 and 1.00 on POD 1 and POD 30 respectively without any statistical significance (Table 3).

**Table 3:** Results of Numeric rating scale among the suture and stapler group.

Numeric Rating Scale (NRS)	Timing of Evaluation	Suture Mean Score (SD)	Stapler Mean Score (SD)	P-Value
	POD1	1.64 (1.76)	1.46 (1.35)	0.596
	POD10	1.31 (1.31)	1.20 (0.91)	0.644
	POD30	1.10 (0.87)	1.00 (0.70)	0.532

Similarly, the mean Numeric Rating Scale (NRS) for pain during removal among sutures and staples was also not significant with a p-value of 0.501.

Type of closure technique and Stony Brook scar evaluation scale

Mean SBSES for the suture group was 3.39 and 2.97, for the stapler group it was 3.06 and 3.02 on POD 1 and POD 30. The results were not comparable (Table 4).

**Table 4:** Results of the Stony Brook Scar Evaluation Scale among the suture and stapler group.

Stony Brook Scar Evaluation Scale (SBSES)	Timing of Evaluation	Suture Mean Score (SD)	Stapler Mean Score (SD)	P-Value
	POD1	3.39 (0.57)	3.06 (0.49)	0.069
	POD10	3.12 (0.54)	3.35 (0.52)	0.046
	POD30	2.97 (0.45)	3.02 (0.33)	0.622

Type of closure technique and readmission, secondary procedures

None of the patients in both groups had required readmission or secondary procedures like wound debridement, re-suturing.

Type of closure and cost

The average cost of a single Ethilon suture (Code 664H) used in our study is INR 163 and of Proximate plus staple (PMW 35) for single-use is INR 1127.

## Discussion

The present study shows a statistically significant difference in the time required to close the LC wound in the stapler group compared to the suture group consistent with other studies. But wound infection rate, pain during removal, and scar results were not statistically significant with the suture group.

The technique of wound closure and meantime of closure

Our study found that the staples have a shorter operative time, three times quicker compared to sutures. In addition to faster closure time, effective use of anesthetic gases by reducing anesthesia time and decreased likelihood of sustaining needlestick injury are the added advantages of using staples.

A study conducted on the pediatric scalp lacerations by Kanegaye et al. revealed that the staples were six times faster than the sutures without observed complications. The scar was cosmetically better and had less pain during removal in the stapler group. Hence, they concluded staple closure was safe, rapid, and cost-effective [7]. Eldrup et al. evaluated 137 patients undergoing thoracic or abdominal surgery and concluded that the time for closure in the stapler group is less than conventional sutures. The closure with staples resulted in the additional expense, as the cost was 47 times higher than that of sutures with Dermalon [8].

The study done by Meiring et al. on 40 patients undergoing laparotomy revealed 80% time saving with staples but concluded that the final cost of the stapler has to be taken into account for its selection [9]. Ranaboldo and Rowe-jones compared skin stapler with subcuticular suture in 48 patients of laparotomy and found that the difference in time was significant, consistent with our study [10].

The technique of wound closure and surgical site infection

The author found no statistical difference in wound infection among the two groups. Tsujinaka et al., in a multicentric study, found no significant difference in wound infection between the subcuticular sutures and skin staples in 1080 patients undergoing skin closure after open gastrointestinal surgery [11]. However, some of the studies found an increased risk of wound infection in the staple groups in different types of operative procedures and varying incision lengths [12,13]. Tuuli et al. found a twofold risk of wound infection in the stapler group in comparison with subcuticular suture in a cesarean wound [12].

Chandrashekar et al. reported wound infection among the stapler group as 38.09% and the suture group was 16% in abdominal wound closure [13]. Basha et al. also found that stapler closure was attributed to an increased risk of wound infection. It led to a decrease in patient satisfaction, but it is not statistically significant to attribute stapler in decreased satisfaction [14].

Type of closure technique and numeric rating scale

Pain during stapler removal has varied opinions among available literature. Some studies reported that the staples have more pain during their removal [15,16]. One of the authors had suggested local application of eutectic mixture of lidocaine and prilocaine (EMLA) cream one hour prior to the removal of skin staples [17]. However, in the present study, there is no difference in pain perceived in both the groups after POD 1 and at the time of removal in accord with the findings of some studies [18-20].

Type of closure technique and Stony Brook scar evaluation scale

The poor and improper technique of suturing can lead to tension at the wound edges, whereas a stapler that can be applied equally on both the wound edges does not increase local tension and ischemia, thus can provide better cosmetic results [21]. Our study did not find any significant difference in post-operative scar status at a one-month follow-up. Cromi et al. found similar results in various skin closure methods in cesarean wounds and found equivalent cosmetic outcomes amongst closure methods [22].

Type of closure and cost

Usually, a single suture was sufficient to close all the laparoscopic port sites which is six times less costly than a skin staple. Our study is consistent with the results of Ranaboldo and Rowe-Jones where staple cost was five times greater than the sutures. Hence their study preferred the use of subcuticular suture [10].

The strengths of the study include the prospective nature of the study, the single surgeon operating and evaluating the cases which removes operator bias, use of validated methods for pain, wound, and scar assessment. Convenient sampling, time-bound cohort study, and small sample size are the limitations of our study. Hence more multicentric randomized studies are required to form a guideline for skin closure in LC wounds.

## Conclusions

The surgical principles are evolving from open techniques to minimally invasive procedures and cosmesis is gaining the utmost importance expecting minimal scars in modern surgical practice. A good cosmetic scar not only gains satisfaction to the patient but also to the surgeon. The mandate to cosmesis is opening research options in search of ideal skin closure methods among conventional sutures, staples, steristrips, clips, and glue adhesives.

The results of our study found that the time taken for the closure of the LC port site wound with staples was three times faster than the suture group and saves operative time. However, there were no comparable results with other parameters studied. We recommend sutures for the LC port site skin closure without adding the additional cost of staples in resource-poor settings.
